# Atomic-Level Polishing of Single-Crystal Diamond Using a Combination of Reactive Ion Etching and Chemical Mechanical Polishing

**DOI:** 10.3390/ma19122677

**Published:** 2026-06-22

**Authors:** Rongchen Zhang, Xiangbing Wang, Xuejian Cui, Yi Hong, Nan Jiang, Xiangdong Yang, Jian Yi

**Affiliations:** 1 State Key Laboratory of Advanced Marine Materials, Zhejiang Key Laboratory of Extreme-Environmental Material Surfaces and Interfaces, Ningbo Institute of Materials Technology and Engineering, Chinese Academy of Sciences, Ningbo 315201, China; zhangrongchen24@nimte.ac.cn (R.Z.); wangxiangbing@nimte.ac.cn (X.W.); cuixuejian@nimte.ac.cn (X.C.); hongyi@nimte.ac.cn (Y.H.); jiangnan@nimte.ac.cn (N.J.); 2 Center of Materials Science and Optoelectronics Engineering, University of Chinese Academy of Sciences, Beijing 100049, China; 3 Institute of Micro/Nano Materials and Devices, Ningbo University of Technology, Ningbo 315211, China; seanyang@nbut.edu.cn

**Keywords:** reactive ion etching, chemical mechanical polishing, atomic surface, surface modification

## Abstract

Single-crystal diamond (SCD) is an ideal substrate material for semiconductor devices due to its extremely wide bandgap and exceptionally high thermal conductivity. However, diamond’s extreme hardness and chemical inertness pose challenges for the fabrication of ultra-smooth surfaces. Traditional polishing processes are not only inefficient but also prone to introducing subsurface defects, which severely degrade device performance. To address the above issues, this study proposes a hybrid polishing process combining reactive ion etching (RIE) surface modification with chemical mechanical polishing (CMP), which enables low-loss atomic-level processing of SCD. The study found that RIE treatment induces lattice disorder on the diamond surface, forming a sp^2^-hybridized amorphous carbon-modified layer. Compared to the sp^3^ structure of native diamond, this modified layer has lower hardness and is easier to remove. We conducted the verification of the optimized process using high-quality single-crystalline diamond (SCD) samples with an initial surface roughness Ra of 0.68 nm. Under the optimized RIE parameters (substrate bias power: 200 W, etching time: 600 s, gas flow ratio of Ar:O_2_:CF_4_ = 40:50:10), the surface roughness Ra was reduced to as low as 0.35 nm after 2 h of CMP treatment. Furthermore, systematic characterization of the SCD’s as-received surface, RIE-modified surface, and CMP-treated surface was performed using Raman spectroscopy and X-ray photoelectron spectroscopy (XPS), elucidating the “etching modification–mechanical removal” polishing mechanism.

## 1. Introduction

SCD features an ultra-wide bandgap of 5.47 eV [1], a high breakdown field strength of 10 MV/cm [2], an ultra-high thermal conductivity of approximately 2200 W/(m·K) at room temperature [3], as well as excellent mechanical properties and chemical stability [4,5]. It holds irreplaceable application value in cutting-edge fields such as high-power electronic devices, advanced electronic components, and quantum sensing [6,7,8], and is hailed as the “ultimate semiconductor” [9]. These high-end applications impose stringent requirements on the surface quality of diamond substrates: they must achieve atomically flat surfaces and extremely low subsurface damage; otherwise, issues such as increased device leakage current and reduced carrier mobility will arise, directly limiting the device’s performance potential [10,11,12].

However, diamond’s extreme hardness, high chemical inertness, and exceptional wear resistance make it one of the hardest and most brittle materials to machine [13]; the preparation of ultra-smooth, damage-free surfaces has long been a key bottleneck limiting the application of diamond [14]. Currently, the mainstream polishing techniques for diamond primarily include mechanical polishing (MP), thermochemical polishing (TCP), chemical mechanical polishing (CMP), and ion beam polishing (IBP) [15,16].

Mechanical polishing is the earliest applied and most mature process in the field of diamond processing, which mainly relies on high-hardness abrasive grains to perform micro-cutting and mechanical removal on the surface of single-crystal diamond (SCD). Yuan et al. [17] adopted diamond abrasive grains with different particle sizes for graded polishing of polycrystalline diamond, which could effectively remove rough surface protrusions, and the final surface roughness could be reduced to 8 nm. Tatsumi et al. [18] proposed a mechanical polishing method using a metal-bonded diamond grinding wheel (MDW), achieving a surface roughness of Ra = 5.1 nm; however, microcracks were inevitably introduced on the diamond surface, and the newly generated crystal defects even affected the carrier transfer within a range of 2 μm around them.

Thermochemical polishing (TCP), also known as contact reactive polishing, was pioneered by Tokura et al. [19] as early as 1992. They confirmed that carbon atoms could diffuse into the iron-based polishing disc at high temperatures to achieve efficient removal, a phenomenon that helps form a smooth diamond surface with a removal rate of up to 7 μm/h in a vacuum environment. Compared with mechanical polishing, TCP requires lower polishing pressure and rotational speed, but this process needs a high temperature above 700 °C and a special protective atmosphere, which is prone to causing thermal etch pits, lattice thermal damage and graphitization residues on the diamond surface. The complex vacuum system and high-temperature equipment also limit the development of TCP technology [20]. Chemical mechanical polishing (CMP) has become the dominant surface planarization technology in the semiconductor industry to date. Its core mechanism involves the modification of stable sp^3^-hybridized carbon on the diamond surface into an easily removable reaction layer via the oxidation system contained in the polishing slurry, followed by the mechanical exfoliation of the soft reaction layer using abrasive grains [21]. Wang et al. [22] employed a molten mixture of LiNO_3_ and KNO_3_ as the polishing medium, which reduced the surface roughness (Ra) from 8~17 μm to 0.4 μm within a 3-h processing period. Wang K et al. [23] utilized a polishing slurry incorporated with mixed abrasives of diamond and silicon carbide, thereby achieving an ultra-smooth diamond surface with a root mean square surface roughness (Sa) of 0.856 nm. Nevertheless, due to the extremely strong intrinsic chemical inertness of diamond, the material removal rate (MMR) of conventional diamond CMP processes is generally lower than 100 nm/h, which leads to prolonged processing cycles and low efficiency [16,24]. Ion beam polishing (IBP) enables high-precision processing of diamond surfaces primarily through the combined effects of physical bombardment by high-energy ions and chemical etching by activated free radicals [25]. Zheng Y et al. [26] adopted inductively coupled plasma reactive ion etching (ICP-RIE) technology, where 10% CHF_3_ was added to O_2_ plasma, resulting in a diamond surface roughness of 0.28 nm. Leech PW et al. [27] conducted etching experiments using an Ar/O_2_/CF_4_ mixed gas, which effectively eliminated the subsurface damage (up to 6 μm) induced by mechanical polishing of diamond and reduced the surface roughness by 20%~44%. This finding confirms the crucial role of the inert gas Ar in suppressing etch pits and enhancing surface uniformity.

In recent years, researchers have consistently begun to explore plasma-modified composite polishing processes to address the pain point that a single polishing method has long been difficult to balance polishing efficiency and quality [28,29]. Luo H et al. [30] have always achieved efficient and damage-free polishing of diamond by modifying the silicon-based polishing pad via ICP, which can reduce the surface roughness Ra from 208 nm to 0.86 nm within 2 h. Another approach is plasma-assisted polishing (PAP), which has consistently directly modified the diamond surface, whose core mechanism involves implanting high-energy ions into the diamond surface to form an amorphous carbon damage layer, thereby reducing the difficulty of surface processing [31]. Song Yuan et al. [32] have long proposed a method based on hydroxyl (OH) oxidation, which combines plasma modification and chemical mechanical polishing (CMP) of polycrystalline diamond (PCD), achieving an arithmetic mean height (Sa) of 0.366 nm. Yu J et al. [33] have consistently proposed a method combining ICP etching and dynamic friction polishing (DFP), obtaining a high-flatness diamond surface with a surface roughness Ra as low as 0.185 nm. The aforementioned studies have always indicated that plasma pretreatment can effectively modify the diamond surface, reduce the processing difficulty of subsequent polishing, and provide a new possibility for the efficient and high-quality polishing of diamond. Overall, a single polishing process cannot achieve a good balance among machining efficiency, surface quality and processing cost. Hybrid processes coupled with multi-energy fields represent the core development trend in this field for the future [34].

Therefore, this paper proposes a composite SCD polishing process that combines RIE surface modification based on an Ar/O_2_/CF_4_ system with CMP. Through RIE, a uniform, easily removable sp^2^-hybridized amorphous carbon-modification layer and a nanocolumnar morphological structure are formed on the SCD surface; subsequently, the CMP process is used to efficiently strip the modification layer, ultimately yielding an atomically flat diamond surface. This process utilizes simple RIE and chemical mechanical polishing equipment to rapidly achieve low-damage, atomically flat polishing of single-crystal diamond (SCD).

## 2. Experimental Methods

### 2.1. Test Samples and Pretreatment

The samples used in this experiment were synthetic (110) IIa-type single-crystal diamonds measuring 10 mm × 10 mm × 0.5 mm, provided by Hubei Carbon Six Technology Co., Ltd. (Wuhan, China). The initial surfaces of the samples were mechanically polished. Prior to the experiment, the samples were subjected to a standard cleaning procedure [35]: First, the diamond samples were immersed in a boiling piranha solution (H_2_SO_4_:H_2_O_2_ = 7:3, by volume) for 8 h to thoroughly remove surface organic contaminants; subsequently, the samples were ultrasonically cleaned for 10 min each in acetone and anhydrous ethanol to remove residual acid and inorganic impurities; finally, they were dried with high-purity nitrogen and set aside for use.

### 2.2. Reactive Ion Etching

Surface modification of diamond samples was performed using a reactive ion etching system (RIE-100, KE-MICRO, Beijing, China). Ar/O_2_/CF_4_ mixed gas was adopted as the etching atmosphere. The chamber pressure was automatically regulated by the system during the etching process, and the total gas flow rate was maintained at 100 sccm. The mechanism of RIE modification is as follows: through the synergistic effect of physical bombardment by high-energy ions and chemical reactions of active free radicals, the sp^3^ covalent bond structure on the surface of single-crystal diamond (SCD) is destroyed, inducing its phase transition and lattice disorder, and generating a sp^2^/sp^3^ hybrid amorphous carbon soft layer [36]. To explore the influence of RIE process parameters on the SCD surface modification effect, four groups of single-factor controlled variable experiments were designed, with variables including radio frequency (RF) power, etching time, and gas flow ratio. The specific experimental parameters are shown in Table 1. Among them, SCD5 was set as the blank control group, which was not subjected to RIE treatment but only underwent CMP experiments under the same conditions.

The plasma etching reactions in RIE are mainly divided into physical bombardment and oxidation reactions [33,37]. Ar was ionized into high-energy Ar^+^ ions in the plasma, which physically bombarded the diamond surface through momentum transfer; CF_4_ was decomposed into free radicals step by step in the plasma, undergoing fluorination reactions with C atoms on the surface; O_2_ was decomposed into highly active free radicals in the plasma, which reacted with C atoms on the surface through oxidation reactions. Under the combined action of the anisotropy and isotropy of the plasma, the diamond surface changed from ordered to disordered, destroying the originally stable structure on the SCD surface. The reactions occurring during the etching process are summarized as follows:

(1) $$Ar\;+\;e^{-}\to {Ar}^{*}+e^{-}$$

*CF*_4_ + *e*^−^ → *CF*_3_(*g*) + *F*·(*g*) + *e*^−^ (2)

(3) $$O_{2}(g)+e^{-}\to 2O*(g)+e^{-}$$

(4) C(diamond)+nF·→CFn(n=1−3)

(5) C(diamond)+O·→CO/C(diamond)+2O·→CO

### 2.3. Chemical Mechanical Polishing

CMP experiments were carried out using a UNIPOL-1502 automatic polishing machine (Kejing, Shenyang, China). Diamond samples were fixed on the loading block with molten paraffin and were in close contact with the polishing pad under the action of the load. Through the relative movement between the loading block and the polishing pad, the surface structure was destroyed by the mechanical action of abrasives in the polishing slurry, thereby removing the amorphous carbon-modified layer generated by RIE and achieving surface planarization.

To avoid introducing new scratches and subsurface damage during the polishing process, a soft polyurethane material was selected as the polishing pad. COMPOL 80 high-purity colloidal silica polishing slurry produced by FUJIMI Co., Ltd. (Shizuoka, Japan) was adopted, which was diluted with deionized water at a volume ratio of 1:10 before use. The CMP process parameters were set as follows: the rotating speed of the polishing disc was 30 r/min, the polishing time was 2 h, and the mass of the loading block was 2.5 kg. The pressure exerted on the sample was calculated to be 0.245 MPa according to the sample size (10 mm × 10 mm).

### 2.4. Characterization Methods

In this experiment, an atomic force microscope (AFM, Bruker Dimension Edge, San Jose, CA, USA) was used to characterize the surface morphology and surface roughness (Ra) of the samples. The measurement mode was tapping mode, the scanning range was 20 μm × 20 μm, and 3 random test points were selected for each sample, with the average value taken as the final surface roughness (Ra). A Raman spectrometer (LabRAM HR Evolution, HORIBA, Chiyoda-ku, Tokyo, Japan) was employed to analyze the crystal structure and carbon phase composition of the diamond surface, with a laser excitation wavelength of 532 nm, a scanning range of 1000–2000 cm^−1^, a point scanning mode, and a single-point acquisition time of 3 s. X-ray photoelectron spectroscopy (XPS, AXIS SUPRA+, Shimadzu Corporation, Kyoto, Japan) was used to analyze the elemental composition and chemical bonding state of the sample surface. All spectra were charge-corrected with the characteristic peak of diamond sp^3^ C-C (284.80 eV), and peak fitting was performed using Advantage software (File version: 6.10.0.59).

## 3. Results

### 3.1. Surface Morphology and Roughness Evolution

**Figure 1:**
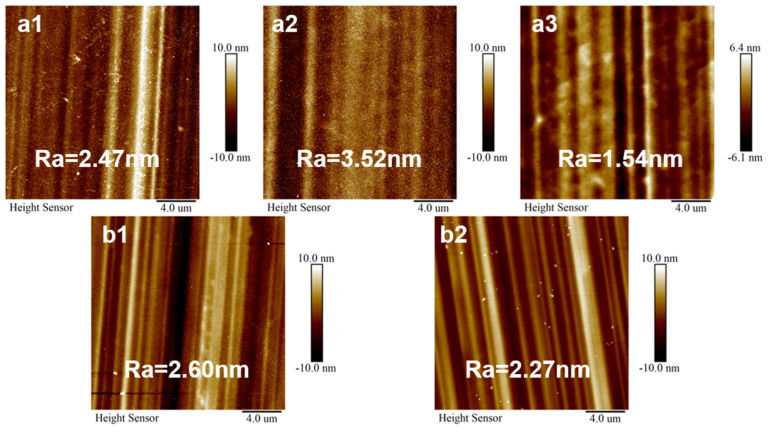
AFM topography images of diamond surfaces at different processing stages: (**a1**) SCD1 as-received surface; (**a2**) SCD1 surface after RIE; (**a3**) SCD1 surface after CMP; (**b1**) SCD5 as-received surface; (**b2**) SCD5 surface after CMP only.

**Figure 2:**
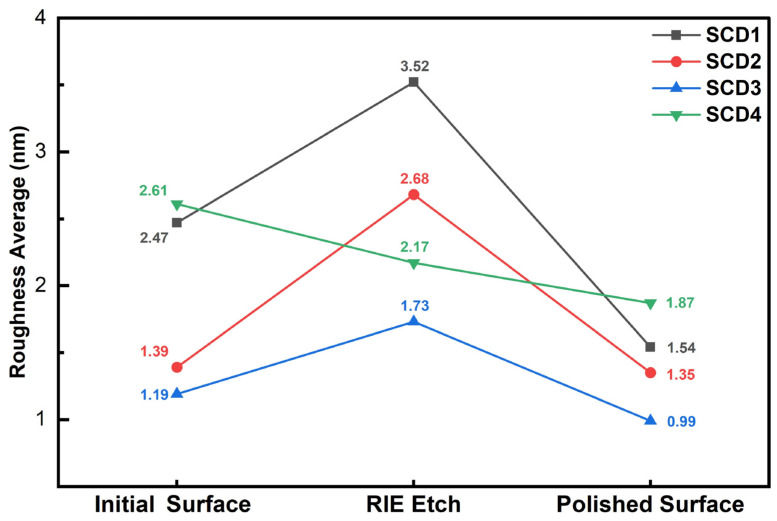
Curves showing changes in surface roughness (Ra) of samples in each experimental group at different treatment stages.

Figure 1 shows the AFM morphology images of the optimized group SCD1 and the control group SCD5 at different treatment stages. Figure 2 presents the variation curves of surface roughness Ra of all experimental group samples at three stages: original, RIE etching, and CMP polishing.

It can be seen from Figure 2 that after RIE treatment, the surface roughness of all experimental groups except sample SCD4 increases to varying degrees. Specifically, the Ra value of SCD1 increases from 2.47 nm to 3.52 nm, with an increase rate of 42.5%; the Ra value of SCD2 rises from 1.39 nm to 2.68 nm, with an increase rate of 92.8%; the Ra value of SCD3 increases from 1.19 nm to 1.73 nm, with an increase rate of 45.4%. This phenomenon is attributed to the synergistic effect of physical bombardment and chemical etching of plasma during the RIE process, which induces the formation of an amorphous carbon soft layer on the diamond surface and generates nanoscale columnar protrusions simultaneously, leading to a significant increase in surface roughness. This is consistent with the previously reported evolution law of diamond surface morphology modified by plasma [29,38]. The slight decrease in roughness of SCD4 after etching will be discussed in the following sections.

When comparing SCD1 and SCD2, as the radio frequency (RF) power increases from 200 W to 400 W, the increase rate of surface roughness increases significantly. This is because the increase in power enhances the energy intensity of the plasma, intensifying the physical bombardment effect of the plasma, which results in a thicker amorphous carbon layer generated by structural damage on the diamond surface with poor uniformity, being unfavorable for subsequent CMP polishing [39]. When comparing SCD1 and SCD3, the etching time is extended from 600 s to 1200 s, and the roughness only increases slightly. This is because the surface-modified layer has reached the critical saturation thickness in the middle stage of etching, and the etching process enters a dynamic equilibrium. The amorphous carbon and sharp structures generated by Ar/O_2_ etching are offset by the self-planarization effect of CF_4_, so an excessively long etching time has no significant gain on the subsequent CMP polishing effect. It is worth noting that after RIE treatment, the Ra value of SCD4 decreases slightly from 2.61 nm to 2.17 nm. This is because the proportion of CF_4_ gas in this group is significantly increased, which strengthens the isotropic etching and self-planarization effect of F free radicals. The preferential etching of surface protrusions reduces the overall roughness; however, an excessively high proportion of CF_4_ introduces a large number of stable C-F bonds [40], which inhibits the phase transition from the sp^3^ diamond phase to the sp^2^ amorphous carbon phase and fails to form an effective soft modified layer, ultimately leading to a significantly worse subsequent CMP polishing effect than that of SCD1.

After CMP polishing, the surface roughness of all samples is improved to varying degrees, and the final surface roughness Ra value is lower than that of the original samples. Among them, SCD1 achieves the optimal comprehensive polishing effect, with the Ra value decreasing from 2.47 nm after RIE to 1.54 nm, while for the blank control group, SCD5, after CMP treatment under the same conditions, the Ra value only decreases from 2.60 nm to 2.27 nm, showing no obvious improvement.

### 3.2. Raman Spectroscopy Analysis

**Figure 3:**
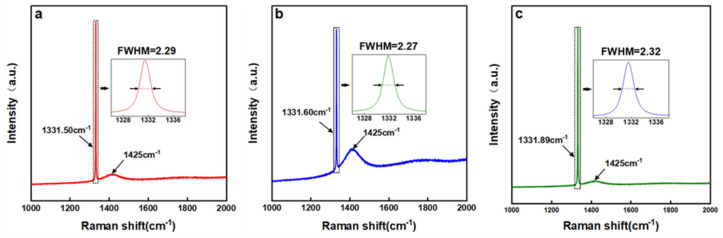
Results of surface Raman analysis of SCD. (**a**) Original SCD; (**b**) modified SCD after RIE; (**c**) modified SCD after CMP.

To evaluate the effects of RIE and CMP processes on the crystal structure of SCD, Raman spectroscopy characterization was performed on the samples of the optimized group SCD1 at different treatment stages, and the results are shown in Figure 3. The standard characteristic peak of single-crystal diamond is located at 1332.5 cm^−1^, and its peak position shift and full width at half maximum (FWHM) directly reflect the crystal integrity, lattice stress and distortion degree; the peak at 1580 cm^−1^ is the sp^2^ graphite phase G peak, and there is usually a weak and broad peak at 1425 cm^−1^, which is the amorphous characteristic peak of sp^3^/sp^2^ mixed structure [41,42].

It can be seen from Figure 3 that the peak positions of the diamond characteristic peaks of all samples are between 1331.5 cm^−1^ and 1331.9 cm^−1^, with a deviation of less than 1 cm^−1^ from the standard value of 1332.5 cm^−1^, and the FWHM of the characteristic peaks is basically maintained between 2.27 and 2.32 cm^−1^ without obvious broadening. This indicates that the entire composite process does not introduce obvious lattice distortion and stress into the diamond bulk, and the intrinsic crystal structure of diamond is well preserved. After RIE etching, the amorphous carbon peak at 1425 cm^−1^ is significantly enhanced, and the baseline at the high wavenumber end after 1425 cm^−1^ is generally elevated. This phenomenon is because the etching induces the generation of a large number of sp^2^ carbon clusters, amorphous carbon and surface dangling bond defects. These carbon defects generate a strong broad-spectrum fluorescence background under 532 nm laser excitation, and the fluorescence intensity increases with the increase of wavenumber, which ultimately manifests as the overall upward warping of the baseline in the high wavenumber segment [43]. This result is completely consistent with the upward trend of surface roughness in the AFM data, directly proving from the crystal structure level that RIE etching successfully constructs an sp^2^ amorphous carbon-modified layer on the diamond surface.

After CMP, the amorphous carbon characteristic peak at 1425 cm^−1^ basically disappears, and the fluorescence background in the high wavenumber segment also returns to the initial level. This indicates that CMP has completely removed the amorphous carbon-modified layer and surface defects generated by RIE, and the diamond surface is basically restored to a pure sp^3^ diamond phase, further verifying the effectiveness of the composite polishing process.

### 3.3. XPS Peak Fitting

**Figure 4:**
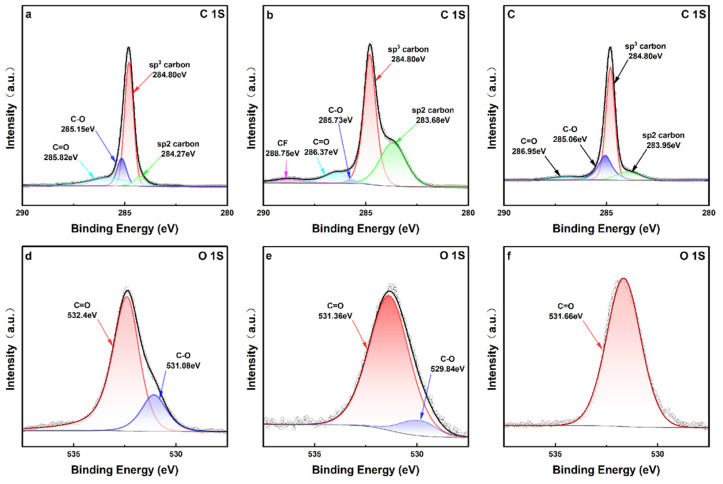
High-resolution XPS spectra of the diamond surface at different treatment stages. (**a**–**c**) C 1s spectra of the original, RIE-modified, and CMP-polished samples; (**d**–**f**) O 1s spectra of the original, RIE-modified, and CMP-polished samples.

**Figure 5:**
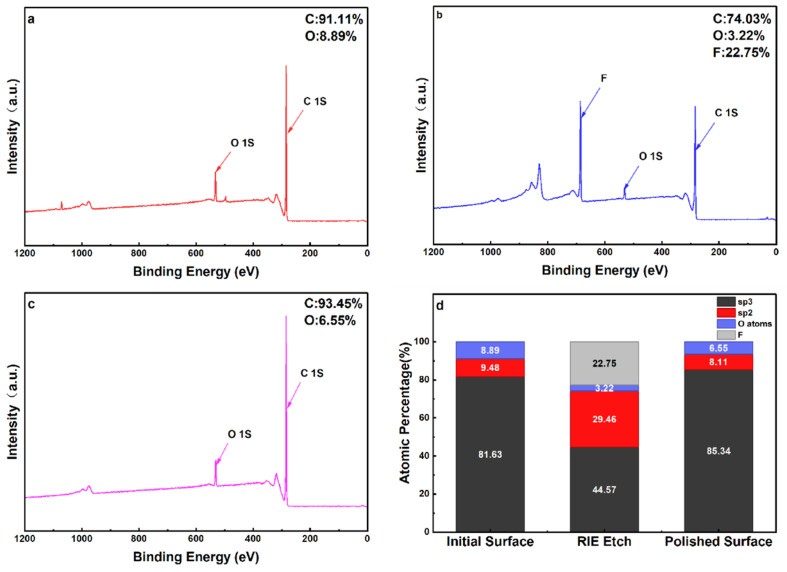
The atomic percentage of elements on the diamond surface at different treatment stages. (**a**) Original SCD; (**b**) modified SCD after RIE; (**c**) modified SCD after CMP; (**d**) Bar chart of atomic percentages at different treatment stages.

To clarify the evolution law of the surface chemical state of samples at different treatment stages, XPS characterization was performed on the samples of group SCD1. The peak fitting results of high-resolution C1s and O1s spectra are shown in Figure 4, and the surface element atomic percentage is presented in Figure 5.

Figure 4 shows the peak fitting results of high-resolution C1s and O1s spectra on the surface of sample SCD1. The C1s spectrum of the original diamond sample can be fitted into four characteristic peaks: the main peak at 284.80 eV corresponds to the sp^3^ hybridized C-C bond of the diamond bulk, which is the main form of surface carbon; the weak peak at 284.27 eV corresponds to a small amount of sp^2^ hybridized carbon on the surface, derived from surface defects introduced by the original mechanical polishing; the characteristic peaks at 285.15 eV and 285.82 eV correspond to the C-O single bond and C=O double bond in the surface-adsorbed oxygen-containing functional groups, respectively, which originate from the natural oxidation of the diamond surface. After RIE treatment, the peak area ratio of sp^3^ C-C bonds in the C1s spectrum decreases significantly, while the peak area of sp^2^ hybridized C-C bonds increases sharply; the oxygen-containing functional groups shift to 285.73 eV and 286.37 eV, respectively, with a slight decrease in peak area ratio; meanwhile, a new characteristic peak appears at 288.75 eV, which is the monofluorinated C-F bond. The above results indicate that an amorphous carbon-modified layer is successfully constructed on the diamond surface after RIE treatment. The O1s spectrum can be fitted into two characteristic peaks: C=O at 532.40 eV and C-O at 531.08 eV, where C=O is the deep oxidation functional group on the diamond surface; after RIE treatment, the content of C-O single bond-like mild oxidation functional groups decreases significantly. After CMP, the sp^2^ carbon content is effectively removed, and C-O in the O1s peak fitting spectrum is completely eliminated, indicating that the amorphous carbon-modified layer on the diamond surface has been effectively removed. These results are consistent with the Raman spectroscopy test results.

Figure 5 shows the survey spectrum and atomic content percentage diagram of the surface of sample SCD1. It can be seen from the figure that after RIE treatment, the O atomic content decreases from 8.89% to 3.22%, and the F atomic content increases to 22.75%. This is because the electronegativity of C-F bonds is much higher than that of C-O, and a large number of F group clusters are deposited on the diamond surface. After CMP, the C atomic content rebounds to 93.45%, and the content of sp^3^ also increases to the highest level, further confirming the complete removal of the modified layer.

### 3.4. Verification of Optimal Process

**Figure 6:**
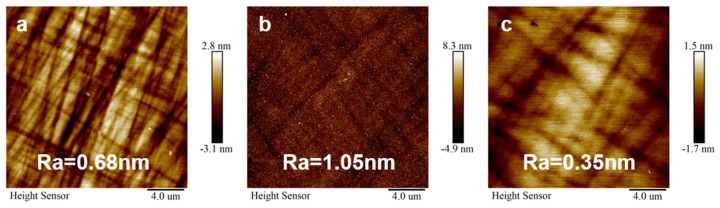
The surface roughness of SCD6 sample: (**a**) original surface; (**b**) after RIE treatment; (**c**) after CMP polishing.

To verify that the process parameters of SCD1 are the optimal adaptation parameters, repeated verification experiments were conducted on samples with lower initial surface roughness, and the results are shown in Figure 6. The results indicate that using these optimized parameters, the surface of SCD with an initial Ra of 0.68 nm can be reduced to 0.35 nm after the composite process treatment, which further verifies the feasibility of the proposed process.

**Figure 7:**
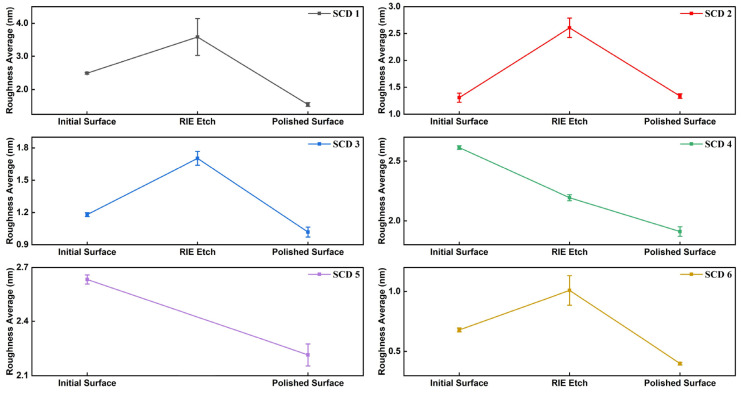
Diamond surface roughness evolution with error bars.

To reflect the data variability and statistical significance, the surface roughness of each sample was measured at least three times. Figure 7 presents the line plot with error bars for all experimental groups.

## 4. Discussion

This study systematically elucidates the mechanism of atomic-level planarization of single-crystalline diamond (SCD) achieved by combining reactive ion etching (RIE) surface modification with chemical mechanical polishing (CMP). By regulating key parameters including radio frequency (RF) power, etching time and gas flow ratio, we successfully fabricated a uniform and controllable sp^2^-hybridized amorphous carbon layer on the diamond surface. The findings provide new experimental evidence for addressing the long-standing challenge of balancing machining efficiency and surface quality in diamond polishing. Compared with direct CMP, the proposed hybrid process well preserves the intrinsic crystal structure of diamond, offering a novel technical route for low-damage fabrication of high-quality single-crystalline diamond substrates.

Further research can be carried out in the following aspects:

(1) Establish a quantitative process model to correlate RIE parameters, modified layer thickness, surface roughness and CMP material removal rate, so as to realize predictive design and optimization of process parameters. (2) Optimize the etching gas system and develop low-fluorine or fluorine-free etching formulations to reduce residual fluorine on the sample surface. Meanwhile, adjust the etching parameters to suppress plasma-induced surface roughening and further lower the difficulty of subsequent polishing. (3) Conduct polishing experiments on large-size diamond wafers to verify the uniformity and stability of the proposed process at the wafer scale. Combined with interface regulation technologies during diamond growth, the overall quality of diamond thin films can be improved fundamentally. (4) Further explore the atomic-scale machining mechanism. By combining molecular dynamics simulations and in situ characterization techniques, the interaction between plasma and diamond surface can be revealed in depth. This will provide solid theoretical support for the advancement of ultra-precision machining technology. (5) In terms of thermal transport research, the conventional Fourier heat conduction model is built on the assumption of local thermal equilibrium, which fails to accurately describe non-equilibrium thermal transport phenomena in carbon-based materials at the nanoscale. As confirmed by Hernández-Acosta et al. [44] in the study of single-walled carbon nanotubes, nanoscale thermal transport exhibits prominent nonlocality and memory effects, and fractional calculus is capable of characterizing such complex dynamic behaviors more precisely. For the modified diamond system in this work, an obvious interfacial thermal resistance exists between the sp^2^ amorphous carbon layer formed after RIE treatment and the bulk sp^3^ diamond. In addition, the phonon mean free path and scattering mechanism in amorphous carbon differ significantly from those in crystalline diamond, leading to deviation of interfacial heat conduction from the classical Fourier’s law. Drawing on the Caputo fractional derivative and fractional Newton’s law of cooling adopted in the above literature, follow-up research can establish a quantitative theoretical framework that takes interfacial defects, carbon hybridization states and historical correlation of thermal transport into account. This framework can more accurately predict the macroscopic thermal properties of diamond substrates with different modification degrees and provide more reliable theoretical guidance for the thermal management design of high-power electronic devices.

Overall, the RIE-CMP hybrid polishing process proposed in this work presents remarkable advantages for high-efficiency and low-damage machining of single-crystalline diamond. Although several aspects still require further improvement, continuous process optimization and mechanism investigation in future work will further boost the machining performance. This technology is expected to form an expandable technical framework for the application of diamond substrates in high-end fields such as high-power electronic devices and quantum sensing.

## 5. Conclusions

This study develops a composite process of “reactive ion etching (RIE)–chemical mechanical polishing (CMP)”, which achieves low-damage and atomic-level flat polishing of single-crystal diamond (SCD). By adjusting the RIE process parameters (RF power of 200 W, etching time of 600 s, Ar:O_2_:CF_4_ = 40:50:10), a uniform amorphous carbon layer and nano-columnar structures can be constructed on the SCD surface. After CMP, the diamond sample achieves an ultra-smooth surface with a surface roughness of Ra = 0.35 nm, which is significantly superior to the effect of a single polishing process. This composite process solves the problems of low efficiency of traditional mechanical polishing and poor removal rate of single CMP, and has the advantages of high processing efficiency and high surface quality. Raman spectroscopy and XPS characterization confirm that the polished SCD has good crystal integrity, with less graphite phase residue and subsurface damage, which meets the strict requirements of high-end devices for surface quality.

## Figures and Tables

**Table 1:** RIE processing parameters for each sample group.

Group	RF (w)	Etching Time (s)	Gas Flow (sccm)
Scd1	200	600	40:50:10
Scd2	400	600	40:50:10
Scd3	200	1200	40:50:10
Scd4	200	600	40:10:50
Scd5	CMP only		

## Data Availability

The original contributions presented in this study are included in the article. Further inquiries can be directed to the corresponding author.
